# Pneumomediastinum as a Manifestation of Granulomatosis with Polyangiitis

**DOI:** 10.1155/2020/3079869

**Published:** 2020-07-30

**Authors:** Ahmed Alhazmi, Mohamed Moulaye Cheikh, Rola Yousef Hassan

**Affiliations:** ^1^Department of Medicine, Division of Rheumatology, King Fahad Armed Forces Hospital, Jeddah, Saudi Arabia; ^2^Doctor Soliman Fakeeh Hospital, Jeddah, Saudi Arabia

## Abstract

A 38-year-old hypertensive male with a smoking history presented to the emergency room with significant hemoptysis, arthritis, and a purpuric rash. Other findings included a dropping hemoglobin and acute kidney injury with microscopic hematuria. The pulmonary computed tomography was significant for alveolar hemorrhage and a rarely reported pneumomediastinum. Along with this constellation of findings, a positive antiproteinase 3 helped to confirm this patient's diagnosis of granulomatosis with polyangiitis. Treatment commenced with sessions of plasma exchange and pulse steroids along with cyclophosphamide infusions. The patient had since improved and made a full recovery. This case report highlights the rarely described pneumomediastinum in association with vasculitides.

## 1. Introduction

Vasculitides are a group of uncommonly seen and diverse inflammatory diseases that affect blood vessels. These conditions may arise unprompted or may be precipitated by an underlying disease. It was first classified by the American College of Rheumatology in 1990 [1], with the Chapel Hill Consensus Conference providing a nomenclature system that more comprehensively covers all types of vasculitides, last updated in 2012 [2]. The most widely known branch of vasculitides is the antineutrophil cytoplasmic antibody-positive vasculitis (ANCA) which encompasses microscopic polyangiitis (MPA), granulomatosis with polyangiitis (GPA), and eosinophilic granulomatosis with polyangiitis (EGPA) [2].

GPA is a necrotizing, granulomatous vasculitis which affects multiple organs including the upper and lower respiratory tract and the kidneys [3]. Common pulmonary radiographic features include nodules, reticulations, segmental atelectasis, widespread opacities associated with air bronchograms, pleural lesions, and rarely hilar lymphadenopathy [4]. The most serious respiratory complication of this disease is alveolar hemorrhage, necessitating aggressive therapy with plasma-pharesis and immunosuppressive drugs [5].

Pneumomediastinum is the existence of air in the mediastinal cavity. This condition can either present spontaneously or secondary to manipulation of the patient's respiratory and digestive systems by trauma, infections, a perforated viscus, or via medical interventions [6]. Data reporting associations between pneumonediastinum and rheumatological diseases are limited, and this association, however, was more commonly pronounced in patients with concomitant interstitial lung disease, as with inflammatory myopathies, systemic lupus erythematosus and systemic sclerosis [7]. There was a case report published in 2014 documenting a presentation of pneumomediastinum in a patient with microscopic polyangitis that was preceded by interstitial pneumonia [8].

## 2. The Case Report

**Figure 1:**
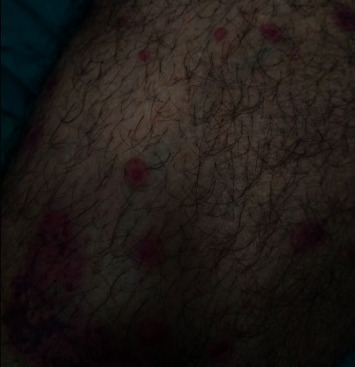
Purpuric rash scattered over the patient's lower limbs.

**Figure 2:**
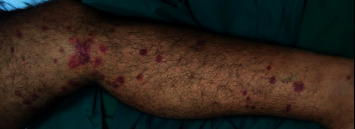
Purpuric rash scattered over the patient's lower limbs.

We are presenting the case of a 38-year-old male who was previously known to be hypertensive and a heavy smoker. He had presented to the emergency room with a 10-day history of productive cough which was associated initially with blood-tinged sputum but had escalated to massive hemoptysis. Further examination revealed the presence of arthritis and a purpuric rash scattered over his lower limbs (Figures 1 and 2). His arthritis was polyarticular, affecting mainly the small joints of the hands (the right first, second, and third proximal interphalangeal joints and the left second proximal interphalangeal joint were tender), and an ultrasound also confirmed grade 1 synovitis in both wrists.

**Figure 3:**
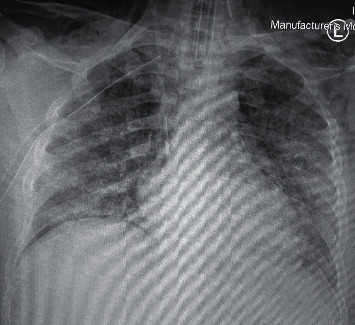
Admission chest X-ray showing bilateral extensive ground glass opacities.

**Figure 4:**
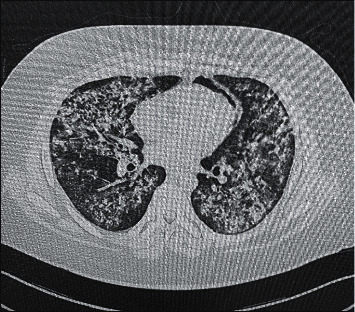
Pretreatment high resolution computed tomography.

The laboratory investigations were significant for a dropping hemoglobin, acute kidney injury, and microscopic hematuria (Table 1). On admission, his chest x-ray showed extensive bilateral ground glass opacities (Figure 3). A pulmonary high-resolution computed tomography (HRCT) was ordered for further assessment and demonstrated evidence of alveolar hemorrhage in the form of diffuse scattered patchy areas of ground glass opacities in both lungs involving all lobes except the apical segments of the upper lung lobes and the subpleuretic areas (Figure 4). There was also a linear lucency noted in the left mediastinum representing pneumomediastinum (Figure 4). A preliminary diagnosis of pulmonary renal vasculitis was made, and a full autoimmune panel was sent.

A few hours after admission, the patient had developed significant hypoxia mandating transfer to the intensive care unit where he was stabilized and promptly started on pulse steroids (methylprednisone 1 gram intravenous daily) and plasma exchange sessions. He required intubation and mechanical ventilation two days after his admission due to deteriorating pulmonary functions and the development of acute respiratory distress syndrome. The patient was started urgently on cyclophosphamide 750 mg intravascular infusion according to the National Institute of Health protocol. He underwent a skin biopsy which gave no evidence of vasculitis. A bronchoalveolar lavage was also performed and grew *Pseudomonas* and *Alicyclobacillus* for which he received colistin and tigecycline for a probable health-care-associated pneumonia (HCAP). After bronchoscopy, the patient had developed right pneumothorax which required the insertion of a chest tube.

The patient's overall condition gradually improved after receiving five sessions of plasma exchange and 5 days of pulse steroids. He was extubated and transferred to the medical ward. His test results came back positive for C antineutrophil cytoplasmic antibodies (cANCA) with a titer of 1 : 20 and anti-proteinase 3 (PR3), whilst P antineutrophil cytoplasmic antibodies (pANCA), myeloperoxidase (MPO), and the antiglomerular basement membrane (anti GBM) were negative (Table 1). A final diagnosis of granulomatosis with polyangitis was made after the exclusion of other causes. The differential included systemic lupus erythematosus which was ruled out based on the absence of supportive clinical features and autoantibodies (Table 1). Other differentials included Goodpasture's syndrome which was deemed less likely with negative anti-GBM and as this condition is not associated with arthritis or purpura. Other small vessel vasculitides such as eosinophilic granulomatosis with polyangitis and microscopic polyangitis were also less likely given that the patient gave no prior history of bronchial asthma, his investigation showed no eosinophilia, and a negative P ANCA/MPO (Table 1).

**Figure 5:**
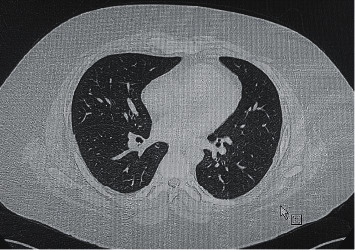
Posttreatment high resolution computed tomography.

The patient had received a second dose of cyclophosphamide during his stay. After a month spent in the hospital, the patient was discharged in a stable condition and given follow-up doses of cyclophosphamide along with tapering steroids in the outpatient setting. A repeated HRCT conducted 12 months after his initial presentation showed complete resolution of his ground glass opacities and pneumomediastinum (Figure 5).

## 3. Discussion

GPA is a multisystemic disease with involvement mainly affecting the respiratory and renal systems [3]. One of the earliest and most impactful papers that closely studied the pattern of GPA lung involvement is the article by Cordier et al. [4]. The authors had characterized the patterns and frequencies of pulmonary findings in 77 patients with biopsy proven GPA. Of this population of patients, 53 “69%” had presented with nodular manifestations, while the occurrence of infiltrates was found in 41 patients “53%.” The incidence of pleural effusions was established in 9 patients “12%,” and only 3 patients “4%” had developed atelectasis. Furthermore, alveolar hemorrhage as a presenting symptom was only reported in 6 patients (8%), and in contrast to our case report findings, pneumomediastinum had not developed in any of these cases.

Pneumomediastinum is the presence of air in the mediastinum. This phenomenon may occur either due to an underlying precipitant or even with the lack thereof. Trauma, infections, and procedures are all known culprits [6]. At the time of our patient's presentation, he had none of these well-described secondary causes. Caceres et al. [6] had further described a different set of factors that could predispose to the development of spontaneous pneumomediastinum. These factors include smoking, inhaled drugs, and airway lung disease such as asthma and chronic obstructive pulmonary disease, as well as idiopathic pulmonary fibrosis. Although smoking was present in our case and might have been a contributing factor to the patient's development of pneumomediastinum, a direct causality relationship cannot be substantiated as the patient had also presented with an active vasculitic lung pathology.

Associations between pneumomediastinum and autoimmune diseases have been scarcely mentioned in the literature. These associations were mainly seen in conditions with a predilection for the development of interstitial lung disease. There have been a few case reports linking pneumomediastinum with systemic sclerosis, systemic lupus erythematosus, and rheumatoid arthritis [9–11]. Pneumomediastinum was also found to be predictive of poor prognosis in patients with dermatomyositis [12]. However, regarding associations with ANCA vasculitis, the only report found was in a patient with a preexisting interstitial pneumonia who developed microscopic polyangitis [8].

Histopathological features of vasculitic pulmonary affection had been delineated by Homma et al. [13] who had studied 31 patients with myeloperoxidase ANCA-associated vasculitis and pulmonary fibrosis. Findings included vasculitic changes in pulmonary arteries and arterioles in three cases each and in capillaries presenting with alveolar hemorrhages in two cases. The characteristic histological features of vasculitis found in pulmonary arterioles were leukocytoclastic angiitis, intimal elastofibrosis with destroyed elastic fibers, and granulomatous angiitis, each in one of the three cases mentioned. Proposed underlying mechanisms for pneumomediastinum on the other hand may include mucosal necrosis in the airways as a result of vasculitic processes at the level of bronchial arterioles, a process that may also manifest with cutaneous vasculopathy, suggest Kono et al. who had studied patients with dermatomyositis and pneumomediastinum [14]. Based on previously laid data, we can extrapolate the pathological pathway of our case, which may have started with mucosal necrosis secondary to GPA vasculitis and subsequent rupture of vulnerable alveolar connective tissue leading to the development of pneumomediastinum.

We hope to further enrich the medical literature with our addition of the first documented association of GPA and pneumomediastinum and to pave the way for more research in this field.

## Figures and Tables

**Table 1:** Laboratory investigations.

	Pretreatment	3 months after treatment
White blood cells	10.9 × 10^9	8.67 × 10^9
Absolute neutrophil count	5.6 × 10^9	8.1 × 10^9
Absolute lymphocyte count	1.9 × 10^9/L	3.9 × 10^9/L
Absolute eosinophil count	0.2 × 10^9/L	0.108 × 10^9/L
Hemoglobin	8.68 g/dL	12.9 g/dL
Platelet	265 × 10^9	334 × 10^9
Creatinine	120 umol/L	114 umol/L
Urea	14 nmol/L	14 umol/L
C-reactive protein	6.5 mg/L	5.5 mg/L
Erythrocyte sedimentation rate	110 MM/HR	17 MM/HR
Urine analysis		
Blood	+3	+1
Red blood cells	+2 (10–34 cells)	Negative
Protein	Negative	Negative
White blood cells	Negative	Negative
Casts	No casts seen	No casts seen
Antinuclear Ab	<1 : 40	
P ANCA	<1 : 20	<1 : 20
C ANCA (indirect immunofluorescence assay)	1 : 20	<1 : 20
PR3	26.2 kU/L	
MPO	1.4 kU/L	
Anti-GBM Ab	0.9 U/mL	

## Data Availability

Access to data can be carried out through hospital records.
